# AKT can modulate the *in vitro* response of HNSCC cells to irreversible EGFR inhibitors

**DOI:** 10.18632/oncotarget.18395

**Published:** 2017-06-07

**Authors:** Renato José Silva-Oliveira, Matias Melendez, Olga Martinho, Maicon F. Zanon, Luciano de Souza Viana, André Lopes Carvalho, Rui Manuel Reis

**Affiliations:** ^1^ Molecular Oncology Research Center, Barretos Cancer Hospital, Barretos, Brazil; ^2^ Life and Health Sciences Research Institute (ICVS), Health Sciences School, University of Minho, Braga, Portugal; ^3^ ICVS/3B’s-PT Government Associate Laboratory, Braga, Portugal; ^4^ Department of Medical Oncology, Barretos Cancer Hospital, Barretos, Brazil

**Keywords:** HNSCC, anti-EGFR, anti- AKT, AKT1, resistance

## Abstract

Epidermal growth factor receptor (EGFR) is overexpressed in up to 90% of head and neck squamous cell carcinoma (HNSCC) tumors. Cetuximab is the first targeted (anti-EGFR) therapy approved for the treatment of HNSCC patients. However, its efficacy is limited due to primary and secondary resistance, and there is no predict biomarkers of response. New generation of EGFR inhibitors with pan HER targeting and irreversible action, such as afatinib and allitinib, represents a significant therapeutic promise. In this study, we intend to compare the potential cytotoxicity of two anti-EGFR inhibitors (afatinib and allitinib) with cetuximab and to identify potential predictive biomarkers of response in a panel of HNSCC cell lines. The mutational analysis in the eight HNSCC cell lines revealed an *EGFR* mutation (p.H773Y) and gene amplification in the HN13 cells. According to the growth inhibition score (GI), allitinib was the most cytotoxic drug, followed by afatinib and finally cetuximab. The higher AKT phosphorylation level was associated with resistance to anti-EGFR agents. Therefore, we further performed drug combinations with anti-AKT agent (MK2206) and *AKT1* gene editing, which demonstrated afatinib and allitinib sensitivity restored. Additionally, *in silico* analysis of TCGA database showed that AKT1 overexpression was present in 14.7% (41/279) of HNSCC cases, and was associated with perineural invasion in advanced stage. In conclusion, allitinib presented a greater cytotoxic profile when compared to afatinib and cetuximab. AKT pathway constitutes a predictive marker of allitinib response and combination with AKT inhibitors could restore response and increase treatment success.

## INTRODUCTION

Head and neck cancer comprises a group of malignancies that occurs mainly within the oral cavity, pharynx, and larynx [1]. This group represents the sixth most common cancer worldwide, with approximately 600,000 new cases diagnosed every year [1]. Squamous cell carcinoma is the most common (90–95%) histologic subtype that arises in the mucous membrane of the upper aerodigestive tract [2]. The primary risk factors associated with head and neck cancer include tobacco use, alcohol consumption, human papillomavirus (HPV) infection (for oropharyngeal cancer), and Epstein-Barr virus (EBV) infection (for nasopharyngeal cancer) [2]. Differences in clinico-pathological, molecular features and prognoses have been reported in HPV-positive and negative HNSCC patients [3, 4].

Alterations in the epidermal growth factor receptor (EGFR) is one of the major events in head and neck squamous cell carcinoma (HNSCC), being overexpressed in up to 90% of patients [5]. Despite the high levels of EGFR overexpression, activating *EGFR* mutations are not frequent and *EGFR* gene amplification is reported in 24-58% of HNSCC [6–8]. Therefore, EGFR has become an important therapeutic target in HNSCC [9]. Several anti-EGFR therapeutic approaches, such as anti-EGFR monoclonal antibodies and EGFR tyrosine kinase inhibitors (EGFR-TKIs), have been developed and some of them approved for the treatment of solid tumors [10, 11]. Cetuximab (Erbitux^®^, Bristol-Myers Squibb; New York, NY), a chimeric monoclonal antibody, which recognizes and binds to the ectodomain of EGFR, preventing its phosphorylation, was one of the first successful drugs in HNSCC [12]. Cetuximab is currently approved in combination with radiation for the treatment of locally advanced HNSCC and in combination with platinum-based chemotherapy for the treatment of recurrent and/or metastatic HNSCC [13]. Nevertheless, cetuximab treatment has shown limited success in HNSCC patients [14, 15]. A recent comprehensive revision reported that cetuximab in combination with platinum-based chemoradiation (CRT) does not lead to an improved outcome survival [16].

More recently, potent pan-HERs inhibitors and irreversible EGFR-TKIs molecules, such as afatinib (Gilotrif®, Boehringer Ingelheim, Inc.) and allitinib (Allist Pharmaceuticals Inc.), have been developed and tested in pre-clinical and clinical trials [17, 18]. Afatinib was specially designed against the *EGFR* secondary mutation T790M and was approved for patients with metastatic non-small cell lung cancer (NSCLC), whose tumors have deletions on epidermal growth factor receptor (EGFR) exon 19 or exon 21 (L858R) substitution mutations [19]. Additionally, in the LUX-Head&Neck 1 trial of second-line afatinib versus methotrexate in recurrent metastatic (R/M) HNSCC patients, a statistically significant improvement in progression-free survival was observed in afatinib compared with methotrexate (2.6 vs. 1.7 months, p=0.003). Therefore, several studies are evaluating afatinib in different scenarios in patients with HNSCC, including an ongoing phase III trial (LUX-Head&Neck 2). Allitinib, is also an irreversible anti-EGFR, with affinity to other EGFR family member proteins (HER2 and HER4) displaying a significant antineoplastic activity *in vitro* and *in vivo* [20]. Moreover, initial phase I clinical trials reported preliminary antineoplastic properties in patients with advanced solids tumor [17].

To better identify patients that could benefit from such targeted therapies, several groups studied potential predictive biomarkers, yet without clear results for HNSCC patients [21, 22]. In colorectal cancer patients, it has been shown that activating mutations of *KRAS* – an EGFR downstream effector - predicts resistance to anti-EGFR monoclonal antibodies therapy in metastatic patients [23, 24]. Patients carrying wild-type *KRAS* showed a two fold better progression-free survival than the mutant ones [23, 24]. Interestingly, our group analyzed the cytotoxic effect of allitinib in a large panel of solid tumor cell lines, and also identified *KRAS* mutation as a biomarker of allitinib resistance [21] Additionally, patients with chemotherapy-refractory metastatic colorectal cancer treated with cetuximab plus chemotherapy, harboring *BRAF*, *NRAS* and *PIK3CA* (exon 20) mutations, had a significantly lower response rate, pointing out the role of alterations in the intracellular pathways for cetuximab response prediction [25, 26]. In HNSCC, *KRAS* mutations are absent or present at very low frequency [4], and markers of cetuximab therapy prediction in HNSCC are still unknown.

Herein, we aimed to do an *in vitro* comparison of the cytotoxicity of two irreversible anti-EGFR inhibitors (afatinib and allitinib) with cetuximab. Moreover, we intend to identify the putative predictive biomarkers of response of these anti-EGFR therapies in HNSCC.

## RESULTS

### Molecular profile of HNSCC cell lines

**Figure 1:**
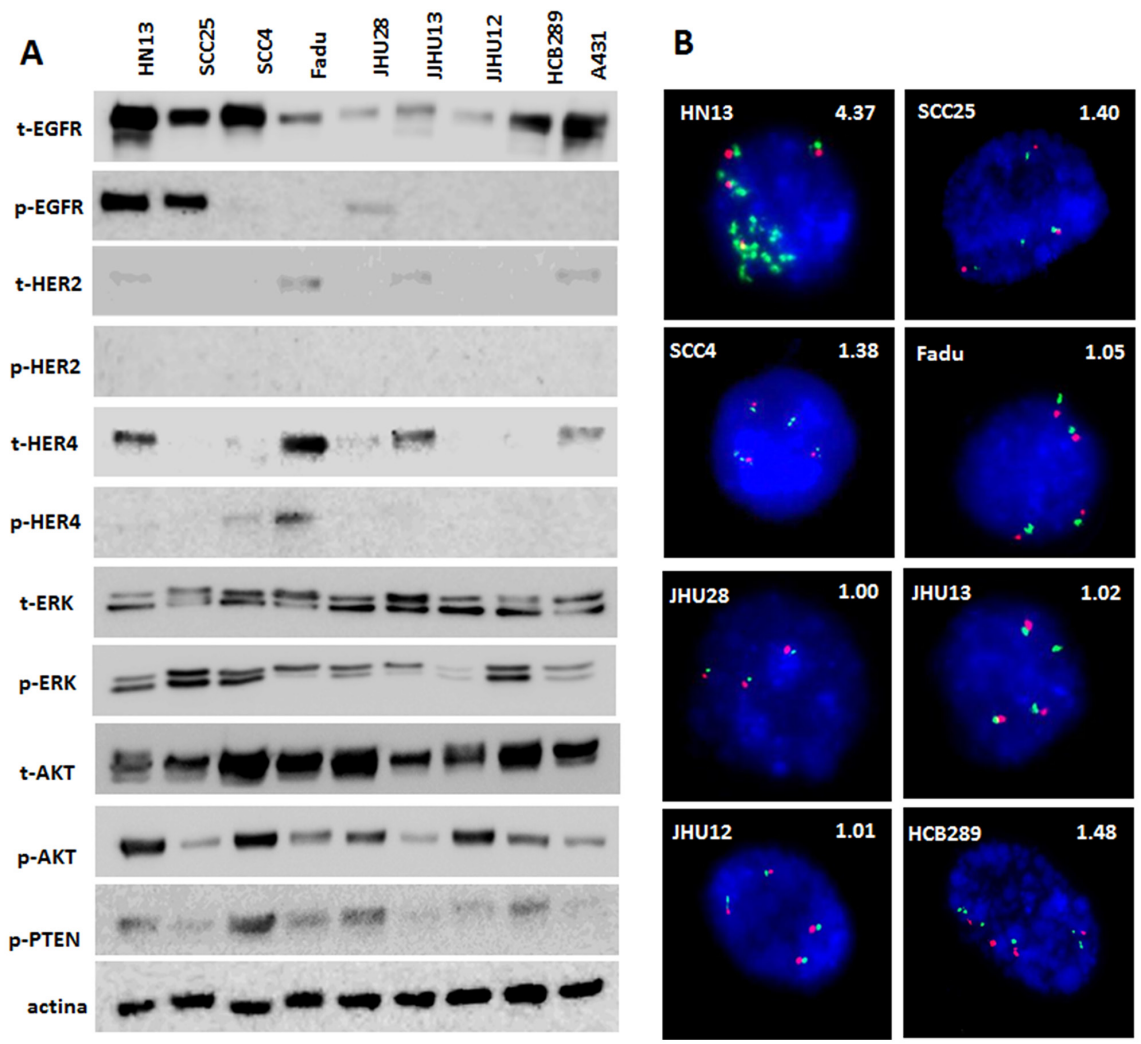
Protein profile and FISH analyses of HNSCC cell lines Total and phosphorylated profile of ErbB family and intracellular pathways, AKT, ERK and total PTEN detected by Western Blot **(A)**. FISH analysis of EGFR gene (green); Centromeric DNA (CEN7) was used as reference (Red). Nuclei were counterstained with DAPI (blue). Magnification: 100X. EGFR/CEN7 ratios are shown above each photograph **(B)**.

The analysis of ErbB family proteins revealed different patterns of expression in HNSCC cell lines. Under basal conditions, HN13, SCC25 and JHU28 showed EGFR phosphorylation, and any of the cell line exhibited HER2 phosphorylation (Figure 1A). Concerning HER4, SCC4 and FADU cell lines displayed HER4 phosphorylation (Figure 1A). We also observed AKT and MAPK intracellular pathways activated in all cell lines, with different levels of phosphorylation. Moreover, 3 out of 7 established cell lines showed absence of total PTEN protein expression, and the HCB289 primary HNSCC cell line, showed low rates of total PTEN expression (Figure 1A).

The mutational status of *EGFR*, *KRAS and NRAS* was previously performed by our group in the established cell lines [21], showing the presence of an *EGFR* missense mutation (p.H773Y) in the HN13 cell line, and a *KRAS* mutation (p.G12S) in the JHU28 cell line (Table 1). Here, the analysis of *BRAF*, *PIK3CA* and *PTEN* genes, did not show additional mutations (Table 1). When analyzing *EGFR* gene amplification, we found that only HN13 cell line have an EGFR:CEN7 signal ratio >4, therefore, being classified as *EGFR*-amplified (Table 1 and Figure 1B).

**Table 1:** HNSCC cell line classification, mutation, amplification status of EGFR response to anti-EGFR therapies

Cell line	Anatomic site	KRAS* mutation	EGFR* mutation	*EGFR amplification*	NRAS* mutation	*BRAF mutation*	*PIK3CA mutation*	*PTEN mutation*	*Cetuximab* μg/mL	*Afatinib (nM)*	*Allitinib (nM)*
**HN13**	Tongue	WT	p.H773Y	Ampli.	WT	WT	WT	WT	> 250	>1000*	>1000
**JHU12**	Oral cavity	WT	WT	No ampli.	WT	WT	WT	WT	> 250	>1000*	>1000
**JHU28**	n.a	p.G12S	WT	No ampli.	WT	WT	WT	WT	> 250	>1000*	>1000
**JHU13**	n.a	WT	WT	No ampli.	WT	WT	WT	WT	> 250	472.60 ± 10.6*	388.94 ± 15.4
**FADU**	Hypopharynx	WT	WT	No ampli.	WT	WT	WT	WT	> 250	774.41 ± 11.7*	384.07 ± 19.0
**SCC25**	Oral cavity	WT	WT	No ampli.	WT	WT	WT	WT	216.85 ± 15.4	224.70 ± 18.4*	207.29 ± 11.6
**SCC4**	Oral cavity	WT	WT	No ampli.	WT	WT	WT	WT	> 250	115.32 ± 9.5*	217.68 ± 16.1
**HCB289**	Oral cavity	WT	WT	No ampli.	WT	WT	WT	WT	> 250	>1000*	845.32 ± 11.5
**A431**	Skin/epidermis	WT	WT	n.a	WT	WT	WT	WT	128.0 ± 2.64	156.92 ± 17.3*	121.31 ± 3.21

n.a: not available; WT: wild type; nM: nanomolar; * mutational status previously described [21].

### Viability effect of cetuximab, afatinib and allitinib in hnscc cell lines

**Figure 2:**
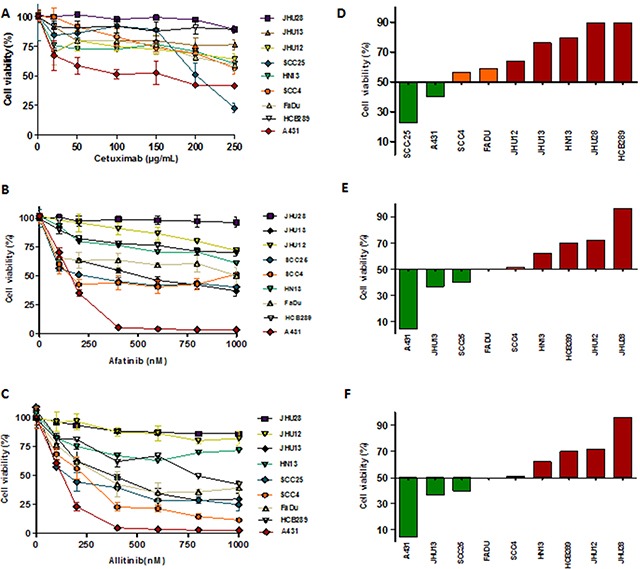
Viability analysis (MTS) of HNSCC cells exposed to different concentrations of cetuximab **(A)**, allitinib **(B)** and afatinib **(C)** for 72 hours. The results were expressed in relation to the DMSO control. GI score of HNSCC cells were calculated for cetuximab **(D)** at 250 μg/mL; allitinib **(E)** and afatinib **(D)** at 1000 nM. HNSCC cells were classified as highly sensitive-HS (green bars), moderate sensitivity-MS (orange bars) and resistant-R (red bars).

Cellular viability analysis to define cytotoxic concentrations of cetuximab (0 to 250 μg/mL) showed that only the positive control of the drug sensitive cell line A431 (IC_50_ = 128.0 ± 2.64 μg/mL) and the HNSCC SCC25 (IC_50_ = 216.85 ± 15.4 μg/mL) cells were responsive (Table 1) (Figure 2A and 2D). It was not possible to calculate the IC_50_ value of cetuximab for all the other cell lines, due to absence of effect at the highest dose used (250 μg/mL) (Table 1). The primary HCB289 cell line was recently established in our Research Center and also exhibited a resistant phenotype to cetuximab (Figure 2D).

Afatinib cytotoxic effect revealed low IC_50_ values for SCC25 (224.70 ± 18.4 nM) and SCC4 (115.32 ± 9.5) cell lines. Regarding allitinib treatment, the lowest IC_50_ values were reached with SCC25 (207.29 ± 11.6 nM), FaDu (384.07 ± 19.0 nM) and JHU13 (388.94 ± 15.4 nM) cell lines (Table 1). In general, JHU28, HN13, JHU12 and HCB289 were the least responsive to both afatinib and allitinib (Table 1).

**Figure 3:**
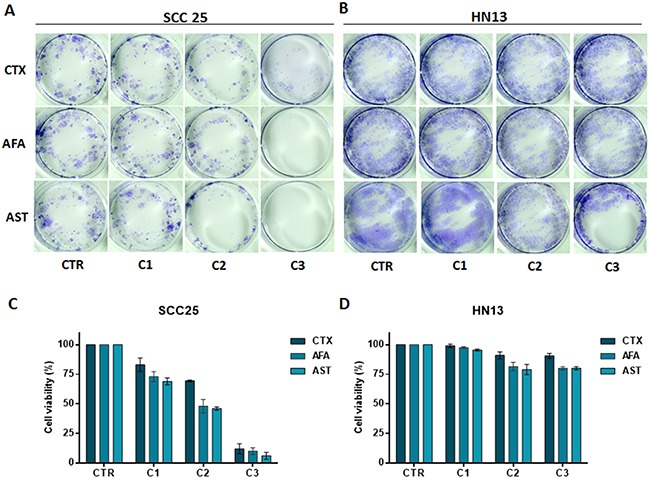
Clonogenic assay of SCC25 **(A)** and HN13 cell lines **(B)**. Cells treated with cetuximab (100, 150 and 250 μg/mL) or afatinib and allitinib (100, 200, and 500 nM). Bars graph represent the relative colony growth of SCC25 **(C)** and HN13 cell line **(D)**. CTR: control; C1, C2 and C3: incremental doses of each anti-EGFR. Data presented as mean of three independent experiments.

Growth inhibition (GI) scores for cetuximab classified the SCC25 cell line as highly sensitive (HS); the SCC4 and FaDu cell lines as moderate sensitive (MS); while the other 5 cell lines were classified as resistant (R) (Figure 2D). For afatinib, GI scores classified JHU13 and SCC25 as HS, FaDu and SCC4 as MS and the other 4 cell lines showed to be resistant (Figure 2E). Finally, for allitinib, the GI scores classified SCC4, SCC25 and JHU13 cell lines as HS; FaDu and HCB289 as MS; and the other 3 cell lines as resistant (Figure 2F). To further validate these findings, the sensitive profile of SCC25 and resistance of HN13 and JHU28 cell lines, was determined by clonogenic assay, which corroborate the MTS results. The SCC25 cell line exhibited lower numbers of viable colonies, showing its sensibility to both anti-EGFR agents (Figure 3A and 3C). At variance, after drugs exposure, the HN13 and JHU28 cell lines did showed a decrease in the number of colony formed (Figure 3B and 3D; Supplementary Figure 1).

**Figure 4:**
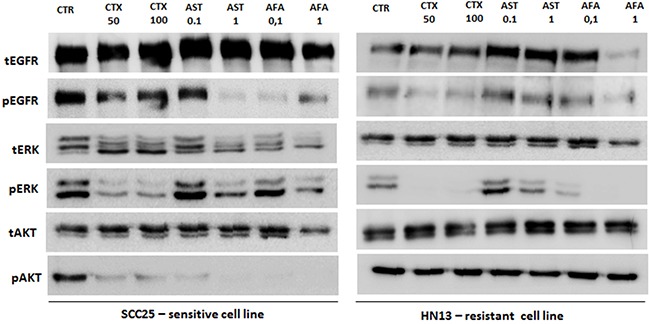
Analysis of EGFR, ERK and AKT total and phosphorylated in SCC25 (highly sensitive) and HN13 cell lines (resistant) by Western Blot EGF ligand was used at 10 ng/mL for 10 minutes. CTR: control; CTX: cetuximab at 50 or 100 μg/mL; AFA: afatinib and AST: allitinib at 0.01 or 0.1 μM.

In order to determine the inhibitory effect of anti-EGFR drugs on EGFR pathways, we selected a responsive (SCC25) and a resistant (HN13) HNSCC cell line. By western-blot analysis, we found a significant reduction of EGFR phosphorylation, mainly following afatinib and allitinib treatment, in SCC25 but not in the HN13 cell line (Figure 4). For the SCC25 cell line, we found that ERK and AKT phosphorylation were reduced when treated with all EGFR inhibitors, being AKT phosphorylation totally abolished when treated with allitinib and afatinib (Figure 4). On the other hand, for HN13 cell line, ERK phosphorylation was completely inhibited upon cetuximab and afatinib exposure, while AKT phosphorylation remained unchanged for all the drug treatments (Figure 4).

Overall, we can observe that the cell lines with lowest AKT activation levels (Figure 1A) also exhibited the highest sensitivity to allitinib and afatinib (A431, SCC25 and JHU13) (Figure 2 and Table 1). In accordance, cell lines with high levels of AKT phosphorylation (such as HN13 and JHU12) depicted less responsive rates to the drugs upon drug treatment (Figure 4). Thus, we hypothesize that AKT activation status could be regulating the response rates of the cells to the drugs.

### Inhibition of AKT pathway can revert resistance to anti-EGFR TKIs

**Figure 5:**
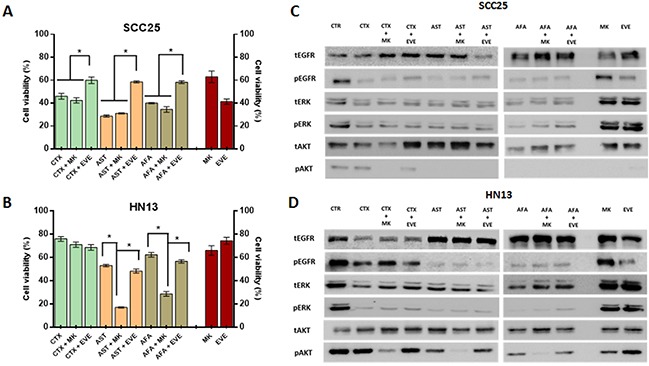
Anti-EGFR therapeutic combinations with intracellular inhibitor MK2206 and everolimus Viability assay (MTS) for SCC25 and HN13 cell lines exposed to cetuximab (250 μg/mL) allitinib or afatinib (1 μM) with or without MK2206 (2.5 μM) and everolimus (EVE) for 72 hours **(A and B)**. CTR: control. Results from combinations were expressed in relation to the DMSO control. Red bars represents cell viability percentage of the Everolimus and MK2206 alone at fixed concentration (2.5 μM). Western Blot analysis of EGFR, ERK and AKT total and phosphorylated in SCC25 (highly sensitive) **(C)** and HN13 cell lines (resistant) **(D)** treated with the AKT inhibitor MK2206 (MK) and mTOR inhibitor everolimus (EVE) at 2.5 μM. SCC25 and HN13 cell lines were exposed to cetuximab (50 μg/mL), allitinib or afatinib (0.01 nM for SCC25 and 0.1 nM for HN13 cell line).

To further test our assumption, we first combined the anti-EGFR agents (cetuximab, afatinib and allitinib) with an AKT inhibitor (MK2206) and mTOR inhibitor (Everolimus) in the same sensitive (SCC25) and resistant (HN13) cell lines (Figure 5). We observed that for SCC25 cell line, the combination with MK2206 showed no differences on cellular viability (Figure 5A), whereas for HN13, the combination of MK2206 with afatinib and allitinib, showed a significant decrease of cellular viability when compared to the effect of the drugs alone (Figure 5B). Unexpectedly, we observed that everolimus led to an increase on cellular viability upon combination with EGFR inhibitors on the SCC25 cell line, while no effect was observed in the HN13 cell line (Figure 5A and 5B).

Additionally, we assessed the potential inhibition of the combined drugs in the intracellular phosphorylation levels (Figure 5C and 5D). In SCC25 cell line (Figure 5C), MK2206 inhibited totally AKT phosphorylation in combination with all anti-EGFR drugs, although in HN13 cell line (Figure 5D) the effect of MK2206 combination showed a total AKT inhibition only for the irreversible inhibitors (afatinib and allitinib), and decreased its levels in combination with cetuximab. We also found that the combination with everolimus did not change the levels of the proteins analyzed (Figure 5D).

Interestingly, we could observe that treatment with MK2206 and everolimus alone does not fully reduce AKT phosphorylation levels in the resistant HN13 cell line at fixed concentration of the 2.5 μM (Figure 5C and 5D) or incremental doses (Supplementary Figure 2).

**Figure 6:**
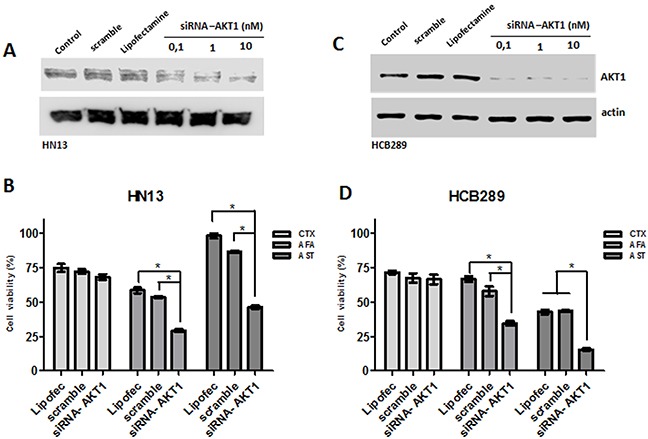
Role of AKT knockdown in cetuximab (CTX) and afatinib (AFA) and allitinib (AST) response in HN13 and HCB289 cell lines **(A and C)** Analysis of AKT knockdown efficiency on HN13 and HCB289 at different AKT siRNA concentrations by Western blot. **(B and D)** Viability assay (MTS) for HN13 and HCB289 cells exposed to Cetuximab (CTX) 250 μg/mL, afatinib (AFA) and allitinib (AST) 1 μM in combination with MK2206 (MK) 2.5 μM or everolimus (EVE) 2.5 μM, for 72 hours. The results from combinations were expressed in relation to the DMSO control. Bars represent viability at 1000 nM concentration.

To further validate the role of AKT in response to the irreversible anti-EGFR TKIs, two resistant phenotype cell lines (HN13, and HCB289) were transiently transfected with *AKT1* siRNA (Figure 6A) and exposed to cetuximab (250 μg/mL), allitinib and afatinib (1000 nM). We observed that both *AKT* knockdown cell lines showed reduction on cellular viability when exposed to allitinib and afatinib, but not when treated with cetuximab (Figure 6B and 6D). These findings corroborate our abovementioned combination results with AKT pharmacological inhibition (MK2206). Additionally, we observed an increased ERK phosphorylation in control experiments conducted in the HN13 AKT1silencing cell line, without anti-EGFR inhibitors (Supplementary Figure 3).

**Figure 7:**
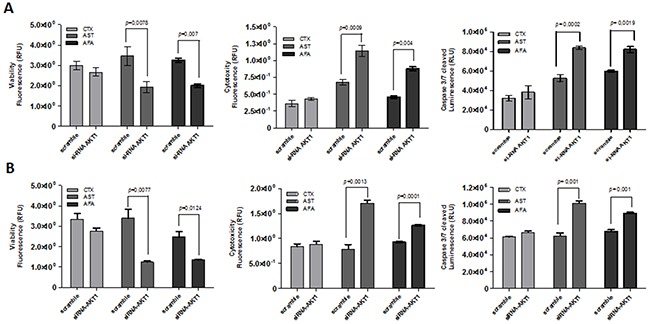
ApoTox-Glo assay of HN13 and HCB289 cell lines transfected with AKT1 siRNA and exposed to cetuximab (CTX) (250 μg/mL) afatinib (AFA) or allitinib (AST) 1 μM, for 24 hours Viability, cytotocity and caspase 3/7 activation of HN13 **(A)** and HCB289 **(B)**.

Furthermore, viability, cytotoxicity and cell death was determined by a fluorescence triple assay in the *AKT1* modulated cell lines exposed to the three EGFR inhibitors. Upon cetuximab treatment, none of the cell lines showed significant cellular changes (Figure 7A and 7B). At variance, following exposure to both afatinib and allitinib both HN13 (Figure 7A) and HCB289 (Figure 7B) cell lines showed a significant decrease on cellular viability, increased cytotoxicity, and increased caspase 3/7 activation. These results demonstrated that AKT could have a key role on HNSCC cell lines response to afatinib and allitinib treatment.

### In silico analysis of ErbB family, AKT and mTOR in HNSCC patients

**Figure 8:**
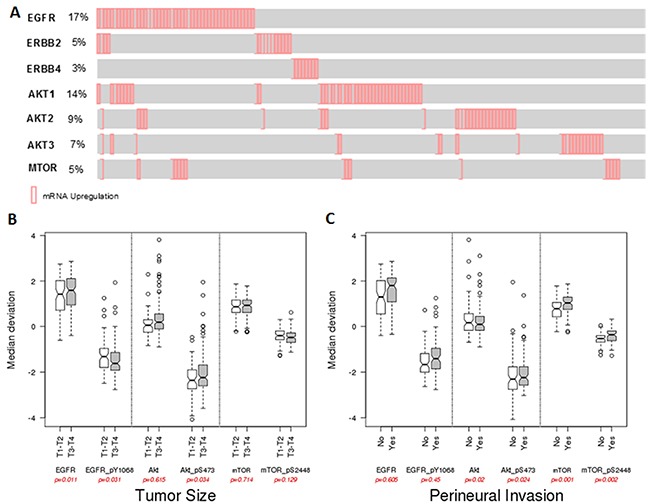
*In silico* analysis from TCGA HNSCC date **(A)** mRNA expression profile of the EGFR, ErbB2, ErbB4, AKT1, AKT2, AKT3 and mTOR. Reverse-phase protein array (RPPA) data and correlations with tumor size **(B)** and perineural invasion **(C)** of HNSCC patients.

Analysis of TCGA database showed that EGFR is overexpressed in 17% (47/279) of the HNSCC patients, and the other ErbB family members, such as ErbB2 (5% - 15/279) and ErbB4 (3% - 8/279) are also overexpressed but at lower frequency. Interestingly, ErbB family overexpression is almost mutually exclusive. We also wondered about the expression of the AKT isoforms and observed that the highest expression levels were found for AKT1 (14.7% - 41/279) (Figure 8A). Moreover, we measured gene expression levels of mTOR that were upregulated in 6% of the patients (16/279). We found that increased EGFR expression and AKT (pS473) phosphorylation were significantly associated with tumor size in T3-T4 stage patients (Figure 8B). Furthermore, AKT1 phosphorylation and total or phosphorylated mTOR were associated with increase on perineural invasion (Figure 8C).

## DISCUSSION

EGFR inhibitors are the most promising agents in HNSCC treatment [8]. Cetuximab, was one of the first drugs developed, and currently the only approved anti-EGFR agent by FDA for HNSCC patients [27]. However, only a fraction of HNSCC patients respond to cetuximab and the critical mechanisms of response remain to be determined [28]. Recently, other classes of EGFR inhibitors have been developed namely irreversible tyrosine kinase inhibitors (irreversible-TKI), such as afatinib and allitinib. Despite great expectations associated with this novel generation of agents, predictive biomarkers of response are still unknown, hampering the establishment of their rational use. In the present study we aimed to compare the efficacy of these three anti-EGFR drugs in a panel of eight HNSCC cell lines, and further identified potential predictive biomarkers of response.

As expected, we found that cetuximab was the least effective agent exhibiting a resistance phenotype with the exception for one (SCC25) of cell lines. At variance, half of the cell lines exhibited a sensitive response to afatinib (two highly-sensitive, and two moderate sensitive), and allitinib was the most efficient with five cell lines displayed a sensitive behavior (three highly-sensitive, and two moderate sensitive). Primary cell cultures are the ideal approach to specifically investigate cytotoxicity, since they better mimic the tumor features. Here, we showed that the primary culture HCB289 was sensitive (HS) only for the irreversible inhibitor allitinib. Overall, these results showed that HNSCC cells are more sensitive to irreversible TKIs, compared to cetuximab.

These results are in agreement with *in vitro* (FADU cell line) and *in vivo* approaches that shown the anti-proliferative effect of afatinib in combination with radiation in a xenograft model [29]. Concerning allitinib, preliminary studies showed a significant antineoplastic activity in *in vitro* and *in vivo* breast, lung and ovarian cancer models, revealing its therapeutic potential [20], yet it was not tested in HNSCC cell lines. Recently, our group demonstrated the potential cytotoxicity effect of allitinib in a large panel of 76 cancer cell lines, including head and neck cancer [21]. Interesting, we this large panel of cell lines evaluated we observed and association of allitinib resistance with the presence of *KRAS* mutations [21]. The mutation screening of our head and neck cell lines showed the presence of *KRAS* (p.G12S) mutation only in the JHU28 cell line, which was classified as resistant to all anti-EGFR therapies, yet overall *KRAS* mutation status, was not associated with allitinib or afitinib response. Additionally, none of the other genes evaluated (*EGFR*, *BRAF*, *NRAS*, *PIK3CA* and *PTEN*) were associated with drug response.

Interestingly, in these HNSCC cell lines, we observed that cell resistance was associated with incapability to decrease AKT phosphorylation levels. AKT1 activation has been reported in gastric [30], prostate cancer [31] and somatic mutation in pleckstrin homology domain (PHD) of AKT1 was reported in human breast, colorectal and ovarian cancers [32]. In addition, increased AKT phosphorylation is related in early event during the papilloma formation in squamous carcinoma *in vivo* model [33] and has been reported as an applicable target for a novel antineoplastic agent in HNSCC tumors [34].

In order to test hypothesis, we initially performed combination of the anti-EGFR agents with AKT (MK2206) and mTOR (everolimus) inhibitors. We showed that combination with MK2206, reduced AKT phosphorylation levels and restored afatinib and allitinib sensitivity to irreversible TKIs in the HN13 resistant cell line. At variance, the combination with everolimus, did not result in total blockage of AKT phosphorylation. Furthermore, everolimus alone increased AKT phosphorylation levels. An *in vitro* study showed that mTOR inhibitor could increase the activation of phospho-AKT and phospho-ERK1/2 by negative feedback loop via the p70S6K–Ras pathway, causing a cross-activation of the Ras–Raf–ERK pathway [35, 36].

Complementary, we knockdown *AKT1* gene in two cell lines, and found that AKT1 silencing restores the response to allitinib and afatinib but not to cetuximab in these cell lines. Overall, our results suggest that irreversible anti-EGFR (TKIs) inhibitors in combination with MK2206 promote a potential benefit for unresponsive cells.

Preclinical studies have showed that *in vivo* SCC1-orthotopic tongue model, treated with AKT inhibitor, showed a reduced tumor size and a preventive effect in metastasis. A recent phase I clinical trial of MK2206 was conducted in patients with advanced solid tumors (NCT00848718). Two of the patients received the MK2206 combination with carboplatin and paclitaxel, docetaxel, or erlotinib and showed a tolerable side effect profile and a complete and partial response [37].

We further evaluated the mRNA expression of the *EGFR*, *ErbB2,ErbB4*, *AKT1*, *AKT2*, *AKT3* and *mTOR* genes in HNCSS patients from TCGA data. As expected, HNSCC patients displayed an upregulation of EGFR mRNA and, we found 5% and 3% of upregulation of other ErbB family, which are target of the new inhibitor allitinib. *AKT1* isoform is predominantly upregulated in HNSCC and besides being associated with radioresistance mechanisms in HNSCC patients [38], more specifically at the AKT(Ser473) residue, it has been used as biomarker to identify HNSCC patients with high risk for treatment failure following radiotherapy [39]. Perineural tumor growth is a frequent event associated with extension described in many cancers including HNSCC [40] and is significantly associated with local recurrence and disease-specific mortality [41], showing elevated incidence rates from 14% to 63.2% in HNSCC [40]. Additionally, we found a statistical correlation AKT1 phosphorylation levels and perineural invasion status, a major important prognostic factor in HNSCC patients. Until now, AKT phosphorylation levels were never associated with perineural invasion in HNSCC patients and this finding enhanced the rational use of the anti-AKT therapy combination.

In conclusion, our study constitutes the first comparative study of the efficacy of two irreversible anti-EGFR inhibitors afatinib and allitinib in HNSCC cell lines. Our results confirm the potent antineoplastic action of allitinib in HNSCC cell lines. Importantly, we identified that persistent AKT activation can play a key role in resistance to the new class of irreversible anti-EGFR drugs, and concluded that therapeutic combinations with AKT inhibitors can revert this phenotype. Further studies are warranted to assess allitinib *in vivo* and clinical benefit for HNSCC patients.

## MATERIALS AND METHODS

### Cell lines and cell culture conditions

A total of eight HNSCC cell lines from different anatomic sites were used in this study. Seven are established and/or commercially available, including HN13, JHU12, JHU13, JHU28 and FADU, as previously described [21]. One cell line, the HCB289, is a primary HNSCC cell line established from a resected primary laryngeal tumor treated at Barretos Cancer Hospital (manuscript in preparation). The A431 (human epithelial carcinoma) was used as positive control of cetuximab efficacy as previous reported [21], and was acquired from Rio de Janeiro Cell Bank. Cell lines were maintained in Dulbecco's modified Eagle's medium (DMEM) or RPMI 1640, containing 10% fetal bovine serum (FBS), 2mM glutamine, 1% penicillin/streptomycin. Cells were incubated in a humidified atmosphere of 5% CO_2_ at 37C°. Unless stated otherwise, all cell culture reagents were purchased from Sigma-Aldrich (St. Louis, USA). All commercial cell lines were authenticated by STR analysis and tested for mycoplasma contamination by PCR [21].

### Pharmacological agents

Monoclonal antibody Cetuximab was purchased from MERK (Darmstadt, Germany); Afatinib (Cat. N° S1011); Allitinib (Cat. N°S2185) and MK2206 (Cat. N°.S1078) were purchased from Selleck Chemicals (Houston, TX) and Everolimus (Cat. N° 07741) was purchased from Sigma Aldrich (Sigma-Aldrich, USA). All drugs were diluted in DMSO at 10 mM and stored at 20°C for future use. DMSO was used as control vehicle at a final concentration of 1% (V/V) in all experiments.

### Cell viability

Cell viability was determined 72 hours after anti-EGFR drug treatments, using the colorimetric CellTiter 96® AQueous One Solution Cell Proliferation Assay (Promega, Madison, WI), according to the manufacturer's instructions and as previous reported [21]. To this end, cells were plated in 96 well plates (maximum 5 × 10^3^/well) in DMEM-10% and allowed to adhere overnight. Subsequently, cells were treated with increased concentrations of cetuximab (20, 50, 100, 150, 200 and 250 μg/mL), afatinib and allitinib (100, 200, 400, 600, 800 and 1000 nM) in DMEM-0.5%. Absorbance was measured using the Varioskan Flash multimode reader (Thermo Scientific, Finland), at 490 nm. Results were normalized with DMSO treated control cells. The IC_50_ values were calculated by nonlinear regression analysis using GraphPad Prism software. Experiments were performed three times in triplicate. Mean growth inhibition (GI) values were calculated at a fixed concentration of 1000 nM (for allitinib and afatinib) or 250 μg/mL (for cetuximab). To determine the cutoff value, A431 cell line was used as a sensibility control for both anti-EGFR therapies. Cell lines were considered as highly sensitive (HS) if GI>60%, moderately sensitive (MS) if GI 40–60% and resistant to if GI<40%, as previously described. [21, 42].

### Clonogenic assay

For clonogenic assays, SCC25, HN13 and JHU28 cell lines were seeded in duplicate into 24-well plates (0.7 × 10^3^ to 1.5 × 10^3^ cells per well) and allowed to adhere overnight in media with 10% of SFB. Subsequently, cells were treated with increased concentrations of cetuximab (100, 150 and 250 μg/mL), afatinib and allitinib (100, 200, and 500 nM) in complete media for 14–17 days. Growth media with each drug was replaced every 3 days. Then, the cells lines were fixed with methanol (1%) and stained with 0.5% crystal violet solution. Colony number was photographed using an optical system Olympus SZX7. Additionally, after extracting crystal violet from the cells lines using 10% of acetic acid, relative colony growth was quantified by absorbance using the Varioskan Flash multimode reader (Thermo Scientific, Finland), at 590 nm. Experiments were performed three times in triplicate.

### Cytotoxicity, proliferation and apoptosis analysis

Apotox-Glo triplex assay (Promega, Madison, USA) was performed in HN13 and HCB289 cell lines, according to manufacturer's instructions, 24 hours after anti-EGFR drug treatments. To this end, cells were plated into 96-well plates at a density of 2 × 10^3^ cells per well and allowed to adhere overnight in DMEM-10%. Subsequently, cells were treated with IC_50_ values of each inhibitor in DMEM-0.5. Luminescence and fluorescence levels were measured using the Varioskan Flash multimode reader (Thermo Scientific, Finland). Results were normalized with DMSO treated control cells. Experiments were performed three times in triplicate.

### Western blot analysis

EGFR inhibition and intracellular signaling pathways were analyzed by western blot in all HNSCC cell lines and in the A431 (sensitive control) cell line. Cells were rinsed in ice-cold PBS then scraped and lysed in lysis buffer (50mM Tris pH7.6–8, 150mM NaCl, 5mM EDTA, 1mM Na3VO4, 10mM NaF, 10mM sodium pyrophosphate, 1% NP-40, and protease cocktail inhibitors). 20 μg of total protein were resolved by 10% SDS-PAGE and transferred to nitrocelulose membranes in TransBlot Turbo transfer (Bio-Rad). Primary antibody incubation was performed for human total EGF Receptor (D38B1), pEGFR-Tyr1068 (D7A5), HER2 (4290), pHER2- Tyr1221/1222 (2243), HER4 (4795), pHER4- Tyr1284 (4757), p44/42 MAPK (137F5); p.p44/42 MAPK-Thr202/Tyr204 (D13.14.4E); AKT(pan) (C67E7); pAKT-Ser473 (D9E); AKT1(C73H10) and β-tubulin (endogenous control), from Cell signaling (Danvers, USA). Both primary antibodies were diluted in TBS-T at 1:1000. After washing with TBS-T, membranes were incubated with anti-rabbit secondary antibody Anti-rabbit (#7074, Cell Signaling Technology) at dilution 1:5000. Immune detection was done with ECL Western Blotting Detection Reagent (GE Healthcare), in automatic ImageQuant mini LAS4000 (GE Healthcare). Experiments were performed three times.

### Fluorescent in situ hybridization (FISH) assay

*EGFR* gene copy number was performed by FISH, using dual color EGFR ZytoLight SPEC EGFR/CEN 7 Dual Color Probe (ZytoVision, Germany). Samples were fixed in methanol/acetic acid (3:1) solution, and washed with 2X SSC buffer at 37°C. Slides were sequentially dehydrated with 70%, 85%, and 100% ethanol and air dried at room temperature. Probes were applied to slides, which were covered and sealed with rubber cement, following manufacturer's protocol. After overnight incubation in a humidified chamber at 37°C, slides were washed with igepal buffer (Sigma-Aldrich, USA) and mounted. Thirty well-defined cells were analyzed for each cell line. Scores were defined as the ratio of EGFR and CEN7, as previously described [7].

### Mutation analysis

*Hotspot regions of EGFR* (exons 18, 19, 20 and 21), *KRAS* (codons 12, 13 and 61) and *NRAS* (codons 12 and 13) were previously reported by our group for the present cell lines [21]. Sequencing of *BRAF* exon 15 (codon 660), *PIK3CA* (exon 9, 21 and 22), and *PTEN* (exon 1-9) genes was performed as described [43, 44] [45]. Briefly, PCR was carried out in a final volume of 15 μl containing 50 ng DNA, 10 μM forward and reverse primers and 7.5 μl HotStar master mix (Qiagen, Hilden, Germany) according to the manufacturer's specifications. Thermal cycling parameters used were an initial denaturation at 96°C for 15 minutes, followed by 40 cycles of 96°C for 45 seconds and 55.5°C for 45 seconds for *BRAF*, 55.5°C for 45 seconds for *PIK3CA* and finally, 52°C for 45 seconds for *PTEN*. For all genes, we used a final extension at 72°C for 10 minutes using a Veriti® 96-Wll Thermal Cycler (Applied Biosystems, Carlsbad, USA). PCR products were purified using EXOSAP-IT (Affymetrix, USB), followed by direct sequencing using an ABI PRISM BigDye XTerminator in conjunction with BigDye XTerminator purification kit (Applied Biosystems). The analyses were performed using the Genetic Analyzer ABI PRISM 3500 and SeqScape version 2.7 software (Applied Biosystems).

### *AKT1* silencing

*AKT1* silencing was performed using TriFECTa-RNAi kit (Integrated DNA Technologies, USA). Each siRNA duplex was transfected using Lipofectamine2000 (Life technologies), in HN13 and HCB289 cells lines, according to the manufacturer's protocol. HN13 cell line was plated into 6-well plates at density of 2.5 × 10^4^ in DMEM-10% and allowed to adhere overnight. Subsequently, cells were exposed to 10 nM of target-specific dicer-substrate siRNAs against *AKT1* gene in reduced serum media Opti-MEM (31985062 - Gibco, Invitrogen) for 5 hours. Subsequently, AKT1 protein expression was measured, 72 hours after transfection, by western blot, using anti-AKT1 antibody (C73H10) from Cell signaling, USA. Knocked-down cells were exposed to the inhibitors above described, and viability was analyzed by MTS and Apotox-Glo triplex assay (Promega, USA).

### In silico analysis of EGFR, AKT1 and mTOR in HNSCC

Reverse-phase protein array (RPPA) data (Level 3) for patients with HNSCC were downloaded from TCGA database (http://cancergenome.nih.gov). A total of 212 tumor samples, with detailed clinical information, were included in this analysis. Both total and phosphorylated values for EGFR, Akt and mTOR data were analyzed. Expression levels (mRNA) of EGFR, AKT isoforms, mTOR were inquired using the cBioPortal (www.cbioportal.org). Cbioportal simple compute the relative expression of an individual gene and tumor to the gene's expression distribution in all samples that are diploid for the gene selected. The returned value indicates the number of standard deviations away from the mean of expression in the reference population (Z-score). This measure is useful to determine whether a gene is up- or down-regulated relative to the normal samples or all other tumor samples. Our z-score threshold was set at ± 2.0.

### Statistical analysis

Single comparisons between the different conditions studied were done using Student's t test, and differences between groups were tested using two-way ANOVA. *In silico* statistical analyses were performed in R Statistical Software (Foundation for Statistical Computing, Vienna, Austria) using Student's t test. All other statistical analyses were conducted using GraphPad Prism version 5. Significance level in all the statistical analyses was set at p < .05

## SUPPLEMENTARY FIGURES


