# Supplementary material for: AKT can modulate the in vitro response of HNSCC cells to irreversible EGFR inhibitors
Source: Oncotarget. 2017 Jun 7;8(32):53288–301. doi: 10.18632/oncotarget.18395 (PMC5581110; doi:10.18632/oncotarget.18395)
Supplement: Supplementary file 1 [file oncotarget-08-53288-s001.pdf]

AKT can modulate the *in vitro* response of HNSCC cells to irreversible EGFR inhibitors

SUPPLEMENTARY MATERIALS

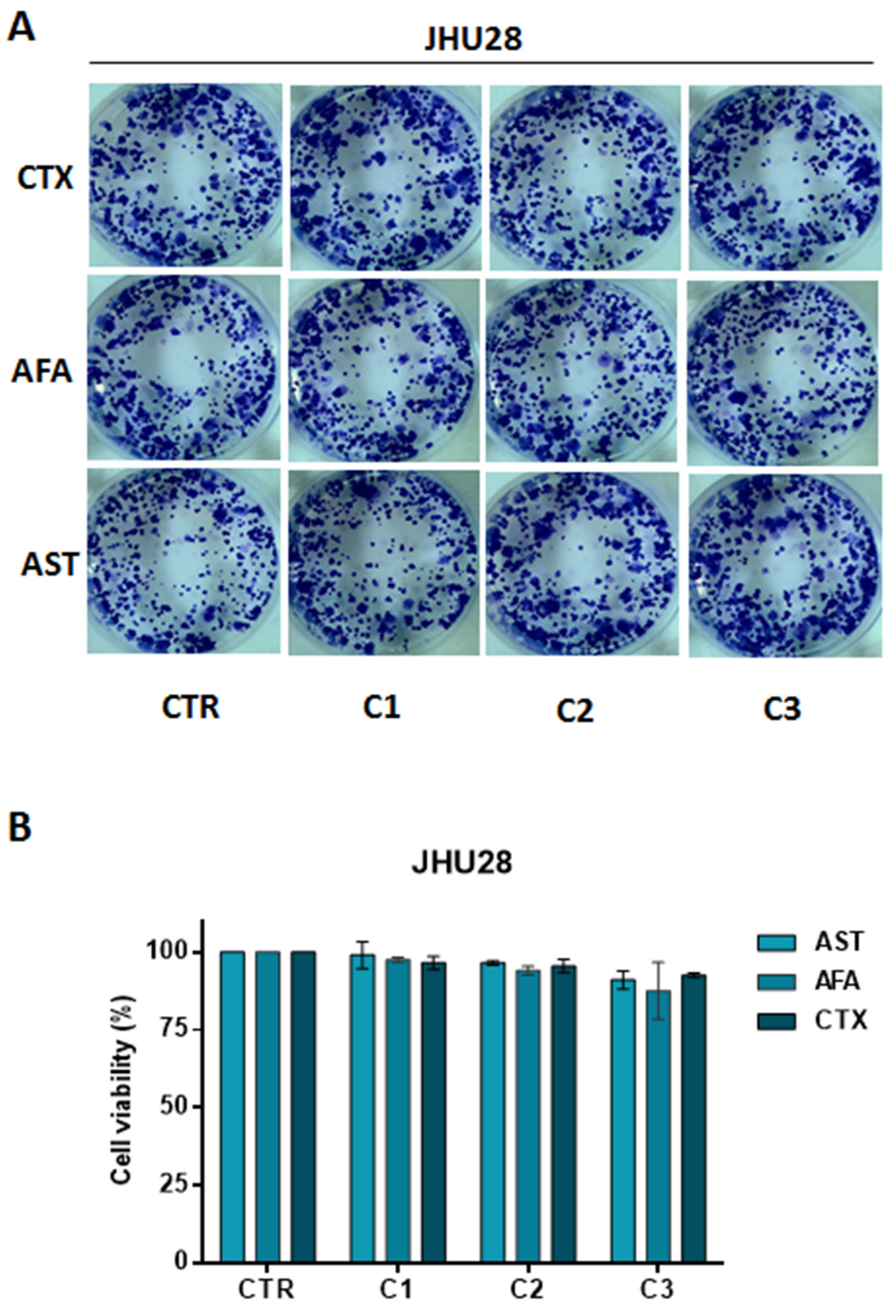

Supplementary Figure 1: Clonogenic assay of JHU28 cell line (A). Cells treated with cetuximab (100, 150 and 250 µg/mL) or afatinib and allitinib (100, 200, and 500 nM). Bars graph represent the relative colony growth of JHU28 cell line **(B)**. Data presented as mean of three independent experiments.

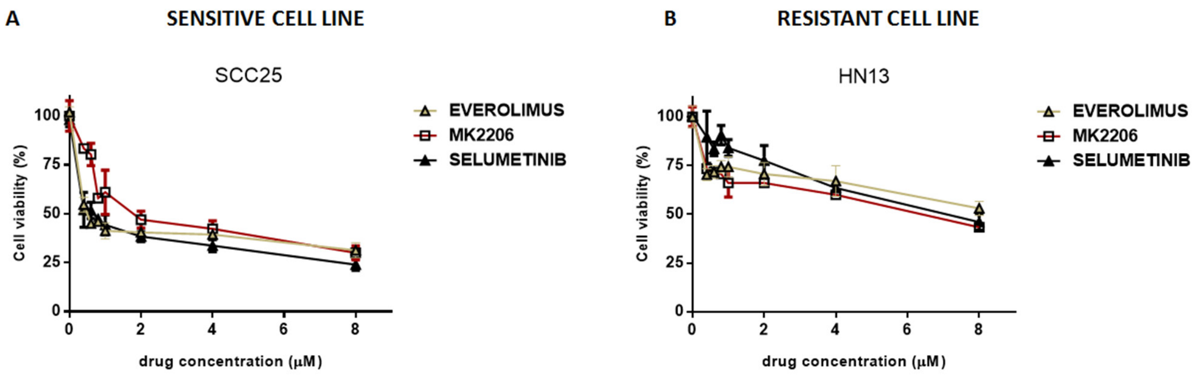

Supplementary Figure 2: Viability analysis (MTS) of sensitive SCC25 (A) and resistance HN13 cells lines (B). Both cell lines were exposed to increased concentrations (0 to 8 µM) of Everolimus, MK2206 and Selumetinib for 72 hours.

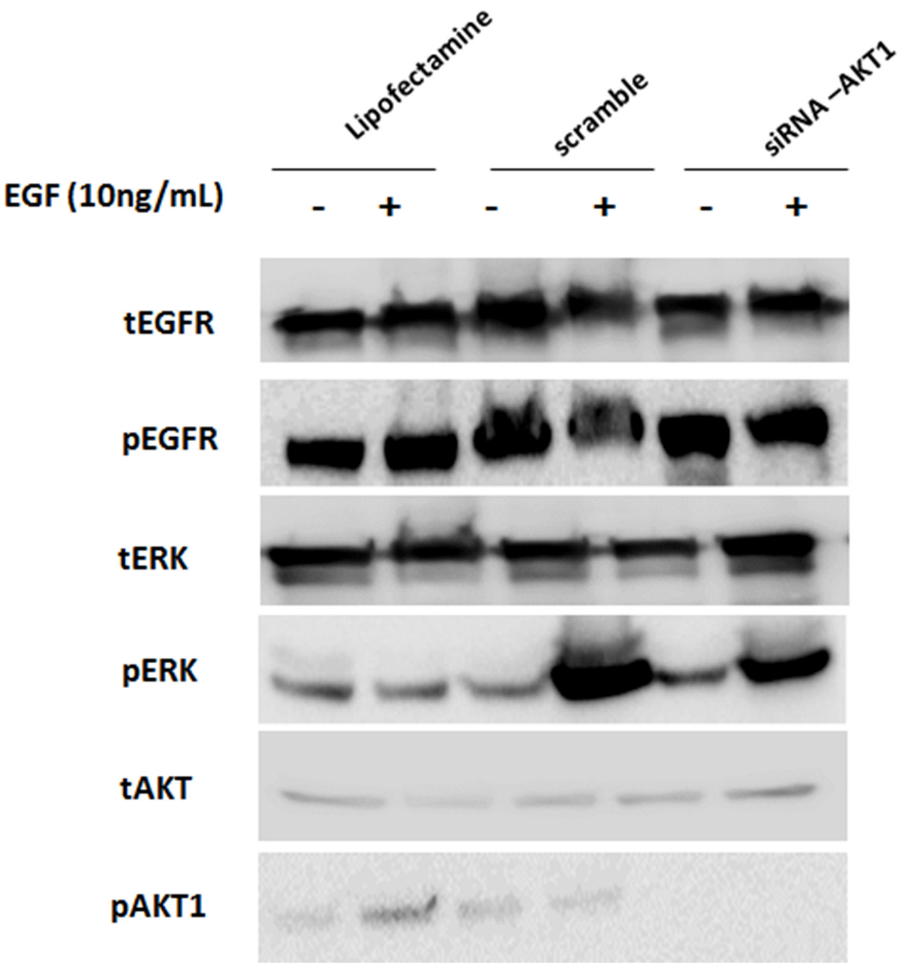

Supplementary Figure 3: Analysis of EGFR, ERK and AKT total and phosphorylated in HN13 (siRNA-AKT1) cell lines by Western Blot. EGF ligand was used at 10 ng/mL for 10 minutes.
